# Italian validation of Arizona Sexual Experience (ASEX) on patients suffering from psychotic spectrum disorders

**DOI:** 10.1192/j.eurpsy.2022.325

**Published:** 2022-09-01

**Authors:** T. Jannini, R. Rossi, V. Sconci, R. Bonanni, F. De Michele, G. Cavallo, A. Siracusano, A. Rossi, G. Di Lorenzo, E. Jannini, G. Ciocca

**Affiliations:** 1 University of Rome Tor Vergata, Department Of Systems Medicine, Rome, Italy; 2 Azienda Sanitaria Locale 01, Department Of Mental Health, L’Aquila, Italy; 3 Sapienza University of Rome, Department Of Dynamic And Clinical Psychology, And Health Studies, Rome, Italy

**Keywords:** ASEX, Psychosis, validation, sexual dysfunction

## Abstract

**Introduction:**

Many forms of mental disorders, especially psychotic disorders are characterized also by a worsening of sexual functioning. Sexual dysfunction has been shown to significantly correlate with a longer duration of untreated psychosis and with heavier psychotic symptomatology.

**Objectives:**

The aim of this study is to validate the Italian version of the Arizona Sexual Experience (ASEX), a very handy and reliable tool to assess sexual dysfunction, in a population of people suffering from psychotic spectrum disorders.

**Methods:**

Seventy-three psychiatric patients were recruited and assessed for mental illness and sexual functioning. We administered the Italian version of ASEX, adequately translated by two expert bilinguals. After 15 days we administered once again the test for test-retest reliability.

**Results:**

Validation of ASEX revealed Cronbach’s coefficients >0.70 in both single items as in the total score. In addition, the test-retest reliability revealed Pearson’s coefficients >0.50 in the various domains. Confirmatory factor analysis revealed good fit indexes for the two factors model of ASEX (SRMR=0.54; CFI=0.974; RMSEA=0.135).

**Conclusions:**

This study represents the first validation in the Italian psychiatric context of a very useful specific tool for the sexual assessment in people suffering from mental illness. Our analysis revealed good psychometric characteristics in terms of confirmatory factor analysis, internal consistency, and test-retest reliability.

**Disclosure:**

No significant relationships.

